# Proving of Saccharum Lactis

**Published:** 1890-03

**Authors:** B. Fincke

**Affiliations:** Brooklyn, N. Y.


### PROVING OF SACCHARUM LACTIS.

B. Fincke, M. D., Brooklyn, N. Y.

(Note.—The following provings have been sent me by Dr. Fincke. He says: "I give you my own literal translation." Dr. Swan's collection of symptoms produced by *Sac-lact.*, published in 1888, will, therefore, have to be somewhat modified and enlarged, according to his original record.—E. W. Berridge.)

(1.) Mrs. T., sixty-odd years, in 1850 or so took of the finest milk-sugar from Wippermann's chemical factory, in Frankfort, triturated three times, according to Hahnemann, and potentiated with distilled water, from the third centesimal trituration to the thirtieth centesimal potency, two drops on her tongue, and observed immediately: Fine drizzling down the back. Burning in the malar bones, going toward the temples and jaw-bones. Severe itching of a liver spot on the right hand. A taste of fresh nuts and a desire for dainties, excited by *Spongia tosta*$^{30}$, disappeared after *Saccharum lactis*$^{30}$, one drop. (See a new proving of *Spongia tosta* in *Amer. Hom. Review*, April, 1859, p. 319.)

(2.) B. F., 1864. April 6th took three drops of *Sach-lact.*$^{1400}$, raised by fluxion from the above 30th, on sugar, and observed strong desire to urinate, with copious discharge.

(3.) Mrs. S., a middle-aged woman; 1864, April 20th, 4.45 p. m., took *Sacchar.-lactis*$^{53m}$, raised by fluxion from the above 30th centesimally, three drops upon a lump of sugar.

8 p. m.—Burning like fire, with sensation of thickness in a strip two fingers wide, from the right frontal eminence to the right side of the vertex for fifteen minutes.

8.15 p. m.—Sudden anxiety, with trembling of the whole body, as of a fright.

Sensation in the region of the heart, as of fire, with a feeling as if it would burst, or also as if something very heavy were lying on the heart, all of which spreads from it to the whole interior and exterior chest.

Longing and depression like homesickness, with heavy breathing; could not sleep before midnight.

It was as if she were young once more, and longed for something which she feared she could not get.

Feeling of mortification and neglect, as if long-forgotten grief were wakened up again.

The heart aches as if it would burst, but she cannot weep.

April 21st.—Dreams not remembered.

The headache from the right frontal eminence to the right side of the vertex, with sensation of a strip of two fingers wide; repeated, and lasting till noon.

Burning and pressure at the heart, with anxiety and apprehension similar to yesterday.

Wretched appearance, and sad expression of the face; the eyes look as if she had wept, though she had not.

Pressure in the stomach, as if she had eaten something indigestible, then heart-burn, with sweetish taste, coming from the stomach, but without water-brash.

Sensation of weakness in the whole body, as after great anxiety and fright.

10 A. M.—Burning in the whole mouth, with a smoothness as after drinking strong liquor.

Fine, spicy taste, as of a certain root.

April 22d.—Sleeplessness after midnight.

The sad mood gradually lessens.

The burning in the chest came on and more coryza.

In the forenoon, a similar condition as yesterday, with the appearance of sadness, but weaker, then burning and painfulness of the left mamma and nipple. (This breast had been sore at a time when she suffered from melancholia for nine months.)

Sensation like ulceration over the right short ribs, anteriorly worse on touching and stooping, and slight swelling between the ribs and skin, not in the liver.

Many dreams of dead people, of small children who are born and die, of brothers who died, but are actually alive yet.

April 23d.—Sensation of ulceration, with slight swelling, worse on touching and stooping, over the short ribs anteriorly, lasting all day till toward evening.

Sleep sound. Feeling well.

April 24th.—4 P. M., painfulness of the right ear-shell, with burning like an ulcer, also on touching.

Stitch over the left eye, backward, continuing over the left eyebrow, that she cannot move the eye, with excruciating pain.

April 25th.—Feeling well.

April 26th.—Good appearance. Feeling well. The habitual sighing has ceased. The vision has improved.

